# What’s new on giant cell tumor of bone

**DOI:** 10.1051/sicotj/2025063

**Published:** 2026-01-22

**Authors:** Shinji Tsukamoto, Costantino Errani, Tessa Balach, Tomas Zamora, Eduardo Ortiz-Cruz, Raja Bhaskara Rajasekaran, Raymond Yau, Tao Ji, Israel Pérez-Muñoz, Francisco Linares, Andrea Angelini, Pietro Ruggieri, Joseph Benevenia, Andreas F. Mavrogenis

**Affiliations:** 1 Department of Orthopaedic Surgery, Nara Medical University 840, Shijo-cho Kashihara-City 634-8521 Nara Japan; 2 Department of Orthopaedic Oncology, IRCCS Istituto Ortopedico Rizzoli Via Pupilli 1 40136 Bologna Italy; 3 Department of Orthopaedic Surgery, University of Chicago 5758 S. Maryland Avenue Chicago 60637 Illinois USA; 4 Department of Orthopaedic Surgery, Pontificia Universidad Catolica de Chile Diagonal Paraguay 362 8330077 Santiago Chile; 5 Orthopaedic Surgery and Traumatology Department, La Paz University Hospital Paseo de la Castellana 261 28046 Madrid Spain; 6 Department of Musculoskeletal Oncology, Ganga Medical Centre & Hospitals Pvt Ltv 313, Mettupalayam Rd, Saibaba Koil Coimbatore 641043 Tamil Nadu India; 7 Department of Orthopaedics & Traumatology, The University of Hong Kong 21 Sassoon Rd Pok Fu Lam Hong Kong PR China; 8 Department of Musculoskeletal Tumor, People’s Hospital, Peking University No. 11 Xizhimen South Street Xicheng District Beijing 100044 PR China; 9 Orthopedics and Orthopedic Surgery Department, Ramon y Cajal University Hospital M-607, Km. 9, 100 Fuencarral-El Pardo 28034 Madrid Spain; 10 Departamento de Ortopedia, Pontificia Universidad Javeriana Carrera 7 No. 40–62 110231 Bogotá Colombia; 11 Department of Orthopedics and Orthopedic Oncology, University of Padova Via Giustiniani 3 35128 Padua Italy; 12 Department of Orthopaedics, Rutgers New Jersey Medical School, Medical Science Building 185 South Orange Avenue Newark 07103 New Jersey USA; 13 First Department of Orthopaedics, National and Kapodistrian University of Athens, School of Medicine 41 Ventouri Street 15562 Holargos Athens Greece

**Keywords:** Giant cell tumor of bone, Denosumab, RFA, TRACP 5b, Curettage, Recurrence

## Abstract

When treating extremities affected by giant cell tumor of bone (GCTB), curettage should be performed to preserve the joint as much as possible in order to obtain a good functional outcome. The local recurrence risk is high following curettage, but new techniques are being developed to reduce local recurrence. We present a review of the literature reporting favorable results of radiofrequency ablation alone in locally recurrent small GCTB. New filling materials are also being developed to prevent non-oncological complications such as arthrosis and fractures. Routine measurement of tartrate-resistant acid phosphatase 5b in serum may be helpful in detecting early instances of local recurrence. For unresectable or metastatic GCTB, there is an urgent need for a new drug that is as effective as denosumab, avoids side effects, and can be administered to pregnant women.

## Introduction

Giant cell tumor of bone (GCTB) is an intermediate-grade primary bone tumor that makes up about 5% of such tumors and has high potential for local invasion [1]. The usual age of onset is around 30 years, and the tumor is usually located at the epiphysis [1], but before epiphyseal closure, tumors can also occur at the metaphysis [2]. Curettage preserves the joint, resulting in better limb function in comparison with en bloc resection, but carries a higher risk of local recurrence [3].

Denosumab received approval in 2013 from the US Food and Drug Administration because it was reported to be safe and effective [4]. Denosumab was reportedly also effective in allowing down-staging, making less invasive surgery an option [5]. However, administering denosumab preoperatively results in bone sclerosis, rendering curettage difficult and further complicating identification of the range of the tumor, which may result in residual tumor. After discontinuation of denosumab, there is the potential of giant cell tumor cells remaining in the sclerotic bone lesions to reactivate [6–9]. Consequently, administering denosumab prior to curettage increases the recurrence rate [6, 7, 10–14]. Therefore, preoperative denosumab is not recommended for GCTB of the extremities where joint preservation is possible. However, in cases where joint preservation is difficult, preoperative denosumab therapy followed by curettage (joint-preserving surgery) may be considered, as re-curettage of recurrent lesions is possible [15]. At present, denosumab is indicated in cases of GCTB that are unresectable or where significant functional impairment is likely after tumor resection [4]. Here, we discuss recent advances in the treatment of GCTB over the past 2–3 years (Table 1).

**Table 1:** Summary of recent advances in the treatment of giant cell tumor of bone.

Type	Study	Subject	Key findings	Comments
Local adjuvant therapy	Jiang [16]	Microwave in situ inactivation in the treatment of GCTB	Thirty patients with GCTB were treated with microwave ablation before curettage, and the local recurrence rate was 0% after an average follow-up of 5 years.	Compared with other techniques, microwave ablation can achieve larger ablation volumes in a shorter time by rapidly inducing coagulation necrosis.
Irrigation solution	Moore [17]	Cytotoxic effects of common irrigation solutions on GCTB	When a human giant cell tumor cell line was immersed in 0.05% chlorhexidine gluconate for 2 min, it exhibited higher cytotoxicity than other liquids.	The use of 0.05% chlorhexidine gluconate solution for irrigation after curettage of GCTB may function as a potential chemical adjuvant.
Filler	Tan [18]	The 3D-printed strut-type prosthesis combined with autograft reconstruction for GCTB of the distal femur	The bone defect after curettage was reconstructed using a 3D-printed strut-type prosthesis, and excellent osseointegration at the bone-prosthesis interface was observed at an average of 4 months.	The 3D-printed strut prostheses in combination with autograft reconstruction exhibited advantages of good biocompatibility, osseointegration ability, and subchondral bone protection.
RFA	Arrigoni [19]	CT-Guided RFA for Management of Surgical Relapses of GCTB	One of five patients who underwent RFA for recurrent lesions after curettage experienced local recurrence, but no complications were observed.	One of the main advantages of RFA is that it provides effective local control with minimal invasion, making it particularly suitable for small, locally recurrent GCTB.
Biomarker	Toda [20]	The diagnostic and prognostic value of tartrate-resistant acid phosphatase isoform (TRACP) 5b for GCTB	The mean TRACP5b change in the group with local recurrence (*n* = 4) was significantly higher than that in the non-recurrence group (*n* = 43) (8.53 and 0.24, respectively, *p* < 0.0001).	Regular measurement of TRACP5b may make it possible to detect local recurrence early.
Systemic therapy	Xu [21]	Efficacy and safety of JMT103 in patients with unresectable or surgically-challenging GCTB	At 3 months post-treatment, the objective tumor response rate was 93%, with hypophosphatemia and hypocalcemia occurring in 65% of patients.	JMT103 represents a potential therapeutic option for GCTB.

GCTB, giant cell tumor of bone; RFA, radiofrequency ablation; TRACP, tartrate-resistant acid phosphatase.

## Local treatment

For tumors of Campanacci stage 1 or 2, the recommended treatment is curettage, in order to preserve the joints and thus ensure a good functional outcome of surgery [22]. As well as simple curettage with a sharp curet, aggressive curettage with a high-speed burr is also recommended [23]. Local adjuvant therapy is also recommended, including administration of ethanol, phenol, an argon beam coagulator, and liquid nitrogen [23]. Recently, good results with microwave ablation have been reported [16]. Jiang et al. conducted a single-center retrospective observational study in which they performed microwave ablation before curettage on 30 patients with GCTB and followed them for an average of 5 years, reporting a 0% local recurrence rate [16]. Microwave ablation has advantages over other techniques, of achieving a larger ablation volume while requiring a shorter procedure time by rapidly inducing coagulation necrosis [24]. Bombardier et al. have performed a variety of local adjuvant therapies followed by histological evaluation in porcine humeri and femoral bone defect models and reported that the mean depth of necrosis was only 0.3 mm in the phenol group, and 0.8 mm in the cement group, while it was 2.5 mm in the argon beam coagulator group, the liquid nitrogen spray group, and the bipolar group [25].

The important thing in curettage is to avoid leaving any undetected tumor. Thus, it is critical to reduce blind spots by enlarging the open window first [22]. There have also been reports describing combining open surgery with scopy in order to find tumors hidden in the blind spots and to prevent tumors from being left behind [26]. Furuta et al. attempted to find residual tumors and prevent tumors from being left behind by employing MRI during curettage [27]. They reported a 100% detection rate of residual tumor by intraoperative MRI [27]. Analysis of recurrence after curettage, in the treatment of GCTB around the knee, found a significant association with a distance of <2 mm between the tumor edge and the articular surface, patient age, and destruction of the posterior cortical bone [28].

After curettage, the tumor is usually washed away, but the best fluid to use as washing liquid was not established. A recent study by Moore et al. investigated which liquids were most effective, by performing in vitro evaluations of the cytotoxic effect on human GCT cell lines exposed individually to sterile water, 0.9% saline, 3% hydrogen peroxide, 70% ethanol, 0.05% chlorhexidine gluconate (CHG), or 0.3% povidone-iodine solution. When the human GCT cell lines were immersed in 0.05% CHG for 2 min they exhibited higher levels of cytotoxicity than when treated with the other washing fluids (*p* < 0.003). As a result, a 0.05% CHG solution may be the best choice for washing after curettage of GCTB, serving as a potential chemical adjuvant [17].

**Figure 1:**
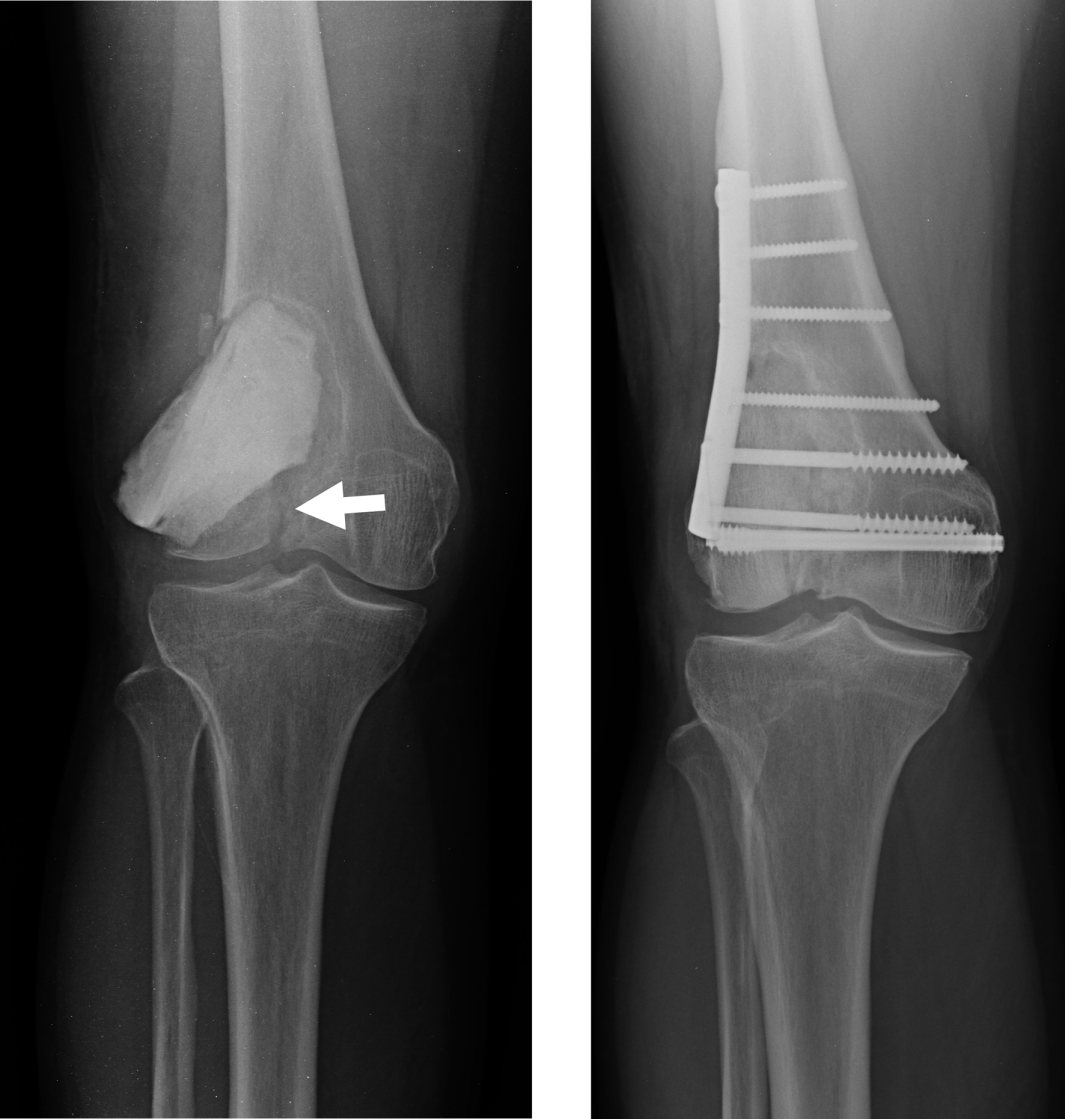
A, Radiography of a new fracture. The arrow indicates the fracture line. B, Radiography 2 years after removal of bone cement, filling of allografts, and osteosynthesis using a plate.

**Figure 2:**
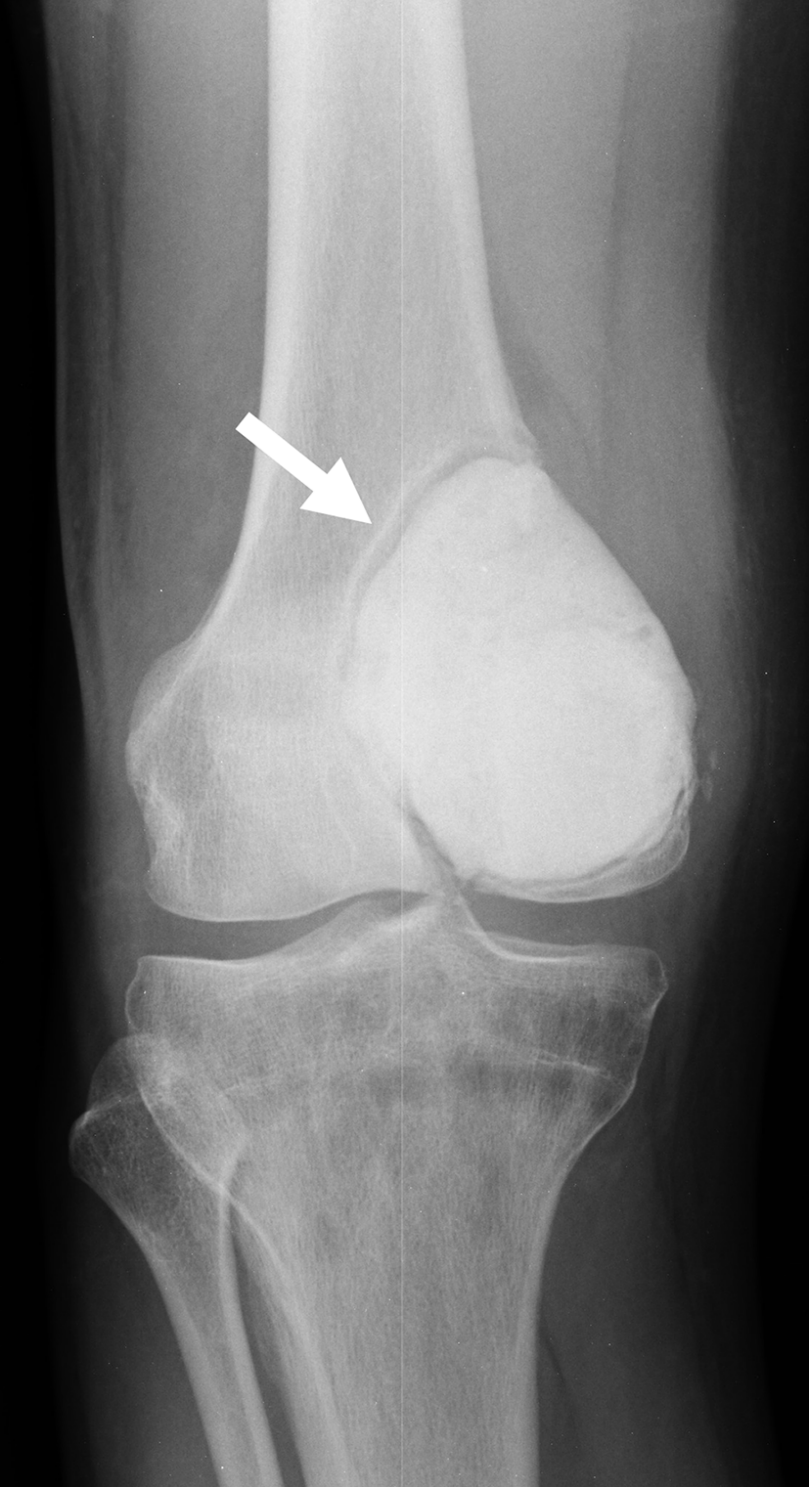
An area with sclerotic rim is seen between the filled bone cement and the bone/subchondral bone (arrow).

**Figure 3:**
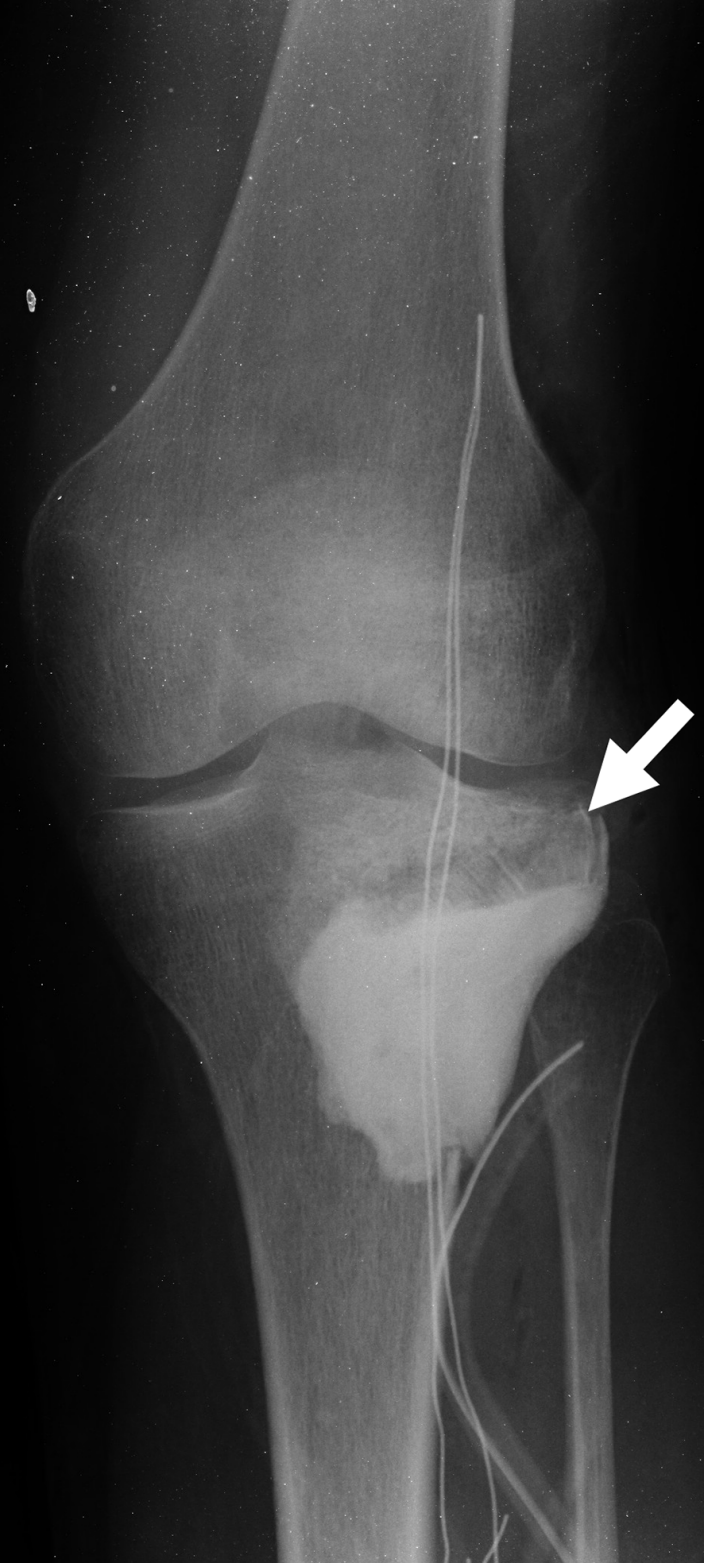
Radiography after curettage followed by filling with bone cement after allograft chip bone filling in the subchondral region. The arrow indicates the allograft chip bone.

When it comes to filling bone defects after removal of a tumor, the materials of choice are hydroxyapatite, β-tricalcium phosphate (β-TCP), bone cement, and allograft. Bone cement has advantages, including the anti-tumor effect of heat, the potential for early weight bearing, and the ease of detecting recurrence on imaging studies [22]. However there are also disadvantages, notably the potential of cement heat to cause cartilage damage when used in subchondral bone, with reports of increased fractures and arthropathy [29, 30] (Figure 1). In addition, because bone cement is stiffer than cartilage or subchondral bone, it results in the concentration of pressure on these already thin tissues [31, 32]. Because bone cement is not biodegradable, it also cannot biologically integrate into the surrounding host bone [33]. In one case, a sclerotic rim was created by increased formation of new trabecular bone, which separated the cement from the surrounding bone and subchondral bone layer [33] (Figure 2), resulting in a reduction in the shock-absorbing capacity of the subchondral bone [33]. Therefore, it is recommended that the subchondral bone should be filled with allograft tip bone and then cemented [29] (Figure 3).

To address this issue of bone cement, a study of 26 GCTB patients by Takeuchi et al. involved filling post-curettage bone defects with calcium phosphate cement. The patients were followed for an average of 87 months to investigate integration of the calcium phosphate cement into the surrounding bone and found that the results were excellent in 22 patients (84.6%), while the outcome was good in three patients (11.5%), and acceptable in one patient (3.8%) [34]. There were instances of local recurrence in three patients (11.5%), while defective remodeling of the cortical bone was observed in 22 patients (84.6%). Evaluation using the Musculoskeletal Tumor Society (MSTS) score gave a mean value of 28.7 (95.7%) [34]. Three patients (3.8%) developed further problems, with one case each of osteoarthritis, chronic synovitis, and fracture, and all were managed conservatively. Thus, it was concluded that calcium phosphate cement provides a biological interface and results in long-term stability without the need for internal fixation [34]. On the other hand, Tan et al. tried an alternative approach and used a 3D-printed strut-type prosthesis together with an autograft to repair bone defects in the distal femur after curettage of a GCTB. They followed nine patients for 30.8 months, on average, after surgery and observed no instances of postoperative complications or local recurrence [18]. Bone union was observed at the graft–host junction in all cases at 3.3 months on average, while at an average of 4.1 months, excellent osseointegration of the bone/prosthesis interface was observed. This approach, employing a 3D-printed strut prosthesis in combination with autograft reconstruction, exhibited advantages of good biocompatibility, osseointegration ability, and subchondral bone protection [18].

**Figure 4:**
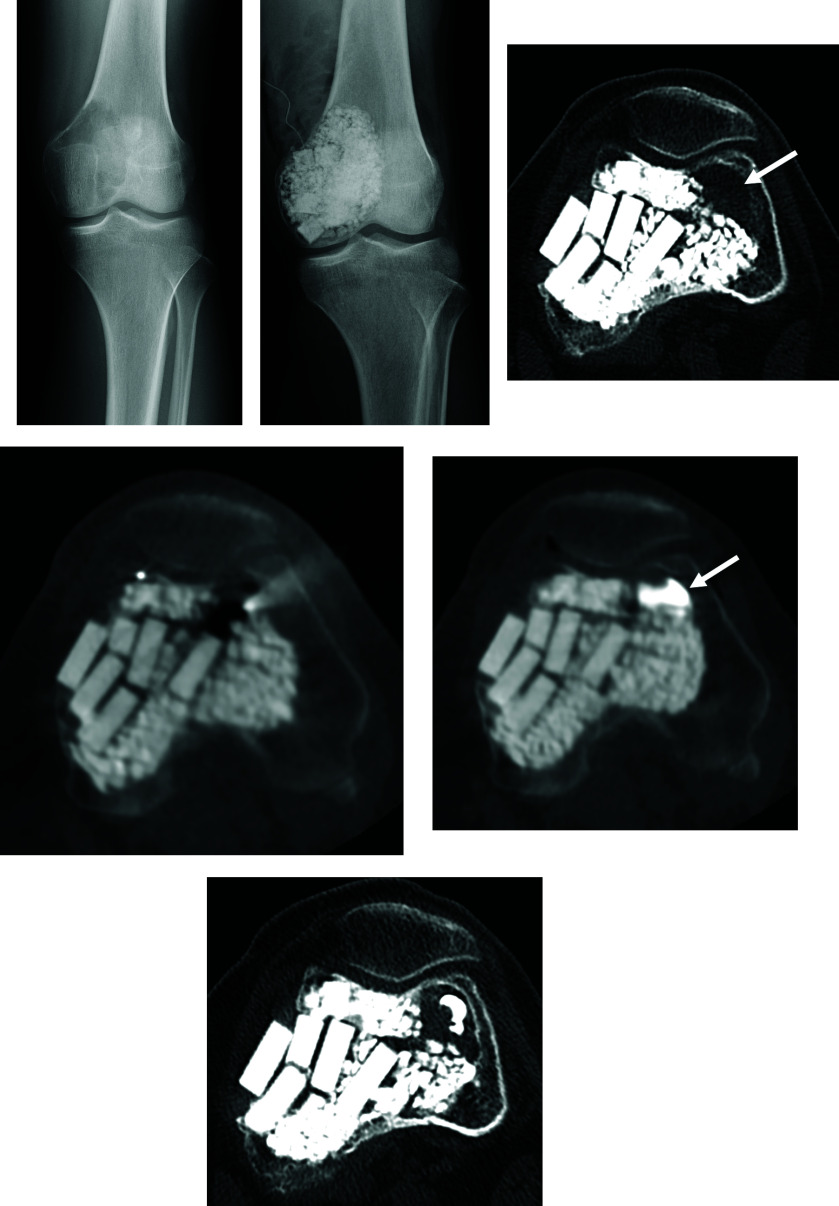
A, Radiography at presentation. B, Radiography after curettage and hydroxyapatite filling. C, CT showed the third local recurrence. After RFA (D), bone cement injection was performed (arrow) (E). F, CT 1 year after RFA showed no local recurrence.

When the extremities are affected, better postoperative function will be achieved if the affected limb is treated by curettage, as far as possible, even for recurrent lesions [35]. Arrigoni et al. conducted a single-center retrospective observational study and reported the outcomes of patients who underwent radiofrequency ablation (RFA) for recurrent lesions after curettage. They found that one of five patients experienced recurrence 4 months after RFA, and subsequently underwent en bloc resection followed by reconstruction with a prosthesis. No complications were recorded [19]. López-Vidaur Franco et al. conducted a single-center retrospective observational study in which they performed RFA on three cases of recurrent GCTB around the knee, and reported that no recurrences were observed after 3–5 years of follow-up [36]. Percutaneous RFA can relieve pain, achieve local control of the lesion, and preserve the function of the joint [19]. One of the main advantages of percutaneous RFA is effective local control with minimal invasion. It is particularly suited for small locally recurrent GCTBs. Furthermore, percutaneous RFA reduces the need for hospitalization because it is performed on an outpatient basis. In the case of small recurrent lesions that are discovered during follow-up after initial curettage, minimally-invasive RFA may be considered first before re-curettage [19] (Figure 4).

Serum tartrate-resistant acid phosphatase (TRACP) 5b has been reported to be a useful marker for detecting recurrence of GCTB [37]. TRACP5b is an osteoclast-derived enzyme and is well known to be an excellent serum marker of bone resorption, reflecting osteoclast count and activity [37]. In one study, patients were classified into groups according to whether or not they experienced local recurrence. The mean value of TRACP 5b in the three patients in the local recurrence group (753 mU/dL) was significantly higher than in the five patients in the non-recurrence group (340.6 mU/dL) [37]. In another study, the mean TRACP5b change in the group with local recurrence (*n* = 4) was significantly higher than that in the non-recurrence group (*n* = 43) (8.53 and 0.24, respectively, *p* < 0.0001) [20]. If local recurrence can be detected early and at a small size by regular measurement of TRACP5b, it may be possible to treat it with RFA alone, resulting in a good functional outcome. If local recurrence is suspected on routine radiographic examinations, additional CT or MRI may be considered [38]. However, CT has the problem of radiation exposure, and MRI is relatively expensive. The prognostic value of alkaline phosphatase (ALP) has been demonstrated in patients with various solid malignancies with bone metastases and osteosarcoma [39–42]. When cancer begins to metastasize to the bone, ALP reflects bone turnover, osteoblast activity, and osteoid formation in the adjacent bone tissue [42]. However, in GCTB, ALP is not elevated (median, 106 U/L (IQR, 63–200)) and cannot be used as a biomarker [43]. TRACP 5b is an inexpensive blood test that does not involve radiation exposure and is therefore considered a particularly useful marker.

A comparison of curettage versus en bloc resection in 51 patients with proximal humeral GCTB was performed by Zhou et al. [44]. A significantly higher recurrence rate was observed in the curettage group (*n* = 23) compared to the en bloc resection group (*n* = 28) (34.8% vs. 3.6%, *p* = 0.007) [44]. Patients were evaluated by MSTS scores, and the values were 26.0 for the group that underwent curettage, 26.0 for the group treated by reverse total shoulder arthroplasty, 20.3 for hemiarthroplasty, and 22.5 for arthrodesis [44]. A lower recurrence rate was achieved by en bloc resection and subsequent reverse shoulder arthroplasty compared to curettage, and the functional outcome scores showed no significant difference compared to curettage for GCTB affecting the proximal humerus [44]. This suggests that reverse total shoulder arthroplasty would be an appropriate initial treatment for GCTB of the proximal humerus [44]. In most cases, GCTBs are marginally resected together with the epiphysis where they are located; as a result, the resected bone is shorter in comparison with the length of bone removed during resection of other primary malignant tumors affecting bone (average tumor size: 6.4 cm) [44]. As a consequence, reverse total shoulder arthroplasty followed by reconstruction is especially suitable in most cases, meaning that the deltoid attachment site and axillary nerve can be preserved.

## Systemic therapy

As an alternative to surgery, denosumab monotherapy may be administered when the high invasiveness associated with en bloc resection is intolerable or when adequate margins would result in unacceptable loss of function [4]. In a Phase 2 study, 532 GCTB patients were treated with denosumab (120 mg once per month) and followed up for a median period of 58.1 months. The study found various side effects of denosumab monotherapy: hypophosphatemia (5%), osteonecrosis of the jaw (3%), anemia (2%), atypical femur fracture (1%), and hypercalcemia (1%) [45]. A retrospective study of progression-free survival, carried out by Jiang et al., reported no statistically-significant difference between dosing intervals of 1 month (*n* = 26) or 3 months (*n* = 14) (*p* = 0.22) [46]. Analysis of tumor control found that longer dosing intervals resulted in similar outcomes compared to the standard dose [46]. On the other hand, Nakata et al. reported that two of three patients who received denosumab every 6 months experienced tumor recurrence during treatment [47]. Therefore, it is recommended that the dosing period be extended to 3 months to reduce the risk of tumor progression.

Denosumab therapy is absolutely contraindicated during pregnancy, and there are no data on the long-term effects of denosumab on the childbearing potential of patients [48]. GCTB is typically diagnosed in women of childbearing age. A systematic review of the literature on GCTB occurring during pregnancy found no clear evidence that pregnancy promotes the growth and aggressiveness of GCTB [49].

One reported case concerned a 31-year-old woman with GCTB of the distal tibia. She was treated with 12 doses of denosumab 120 mg (9 months) followed by zoledronic acid 4 mg for 6 doses (3 years), and the tumor shrank, with no serious adverse events. At 41 months after discontinuation of denosumab, and 10 months after the final infusion of zoledronic acid, the patient remained in stable clinical remission with no serious adverse events. It is speculated that the high uptake of zoledronic acid into the lesion after ossification of the tumor by denosumab resulted in a high antitumor effect [50].

A multicenter, single-arm, open-label, phase Ib/II study was carried out to evaluate the safety and efficacy of JMT103, an antibody targeting receptor-activator of nuclear factor-kappa ß ligand, in patients with unresectable or surgically-treated GCTB [21]. A total of 135 patients were treated with JMT103 (2 mg/kg) administered subcutaneously every 4 weeks with loading doses on days 8 and 15. At 3 months post-treatment, the objective tumor response rate was 93.3%, with hypophosphatemia and hypocalcemia occurring in 65% of patients. Thus, JMT103 represents a potential therapeutic option for GCTB [21]. JMT103 is a fully humanized monoclonal antibody and has the same Fab arm as denosumab, while the Fc-terminal is changed from IgG2 to IgG4 [21]. Previous studies have revealed that human IgG2 monoclonal antibodies undergo disulfide scrambling [51, 52] and this may play a part in the heterogeneity of therapeutic monoclonal antibodies produced [53]. IgG4, a “blocking antibody,” may have a better safety profile than IgG2 because it does not form large complexes that may induce effector function [54]. A phase I study of JMT103 in patients with metastatic bone lesions also found that it resulted in rapid, significant, and sustained suppression of bone resorption biomarkers [55].

## Conclusion

New techniques and the latest findings have been reported to reduce local recurrence after curettage of GCTB. New filling materials that prevent non-oncological complications such as arthrosis and fractures due to bone cement have also been reported. Favorable results have been reported with RFA monotherapy for small locally recurrent GCTB. Routine measurement of serum TRACP5b may be useful for early detection of local recurrence. There is a need to develop a drug that is as effective as denosumab, avoiding side effects, and can be administered to pregnant women. Multicenter prospective studies and integration of molecular and imaging biomarkers are needed to define the next generation of personalized GCTB management.

## Data Availability

Data is not available for this article.
